# Risk of suicide attempt and suicide in young adult refugees compared to their Swedish-born peers: a register-based cohort study

**DOI:** 10.1007/s00127-021-02099-5

**Published:** 2021-04-29

**Authors:** Gerdur Geirsdottir, Ellenor Mittendorfer-Rutz, Ridwanul Amin

**Affiliations:** Division of Insurance Medicine, Department of Clinical Neuroscience, Karolinska Institutet, 17177 Stockholm, Sweden

**Keywords:** Suicide, Suicide attempt, Refugee, Minor, Unaccompanied, Migration

## Abstract

**Purpose:**

Refugees, especially minors, who often have experienced traumatic events, are a vulnerable group regarding poor mental health. Little is known, however, of their risk of suicidal behaviour as young adults. We aimed to investigate the risk of suicidal behaviour for young adult refugees who migrated as minors. The moderating role of education and history of mental disorders in this association was also investigated.

**Methods:**

In this register linkage study, all 19–30-year-old Swedish-born (*n* = 1,149,855) and refugees (*n* = 51,098) residing in Sweden on December 31st, 2009 were included. The follow-up period covered 2010–2016. Cox models were used to calculate hazard ratios (HRs) with 95% confidence intervals (CIs). The multivariate models were adjusted for socio-demographic, labour market marginalisation and health-related factors.

**Results:**

Compared to Swedish-born, the risk of suicide attempt was lower for all refugees (HR 0.78, 95% CI 0.70–0.87), and accompanied refugee minors (HR 0.77, 95% CI 0.69–0.87), but estimates did not differ for unaccompanied refugee minors (HR 0.83, 95% CI 0.62–1.10). Low education and previous mental disorders increased the risk of suicide attempt in both refugees and Swedish-born, with lower excess risks in refugees. Findings for suicide were similar to those of suicide attempt.

**Conclusion:**

Young adult refugees have a lower risk of suicidal behaviour than their Swedish-born peers, even if they have low educational level or have mental disorders. Young refugees who entered Sweden unaccompanied do not seem to be equally protected and need specific attention.

**Supplementary Information:**

The online version contains supplementary material available at 10.1007/s00127-021-02099-5.

## Background

The number of forcibly displaced people worldwide is currently record high, and it is believed that 50% of all refugees are refugee children [1]. Such a pattern of forced migration in recent decades has brought about demographical changes in many European countries, including Sweden. The arrival of unaccompanied refugee minors to Sweden started to increase from 2005 [2]. In 2015, approximately 70,000 refugee minors arrived in Sweden, where about 50% were unaccompanied refugee minors [3].

The entire process of immigration can be stressful for refugees and especially refugee minors, both before-, during- and post-migration [4]. Refugees are likely to have faced traumatic events in their home country, such as physical harm or having to leave their family behind [4]. Traumatic pre-migration events like war and poverty [5], but even violence-related events such as torture, death of family members and rape, are strongly associated with psychological distress among refugees [6]. Incidents like separation and sexual abuse during migration can also be traumatising and affect the risk for mental disorders as well as the lack of integration, access to education, and social networks after migration [7, 8].

Evidently, a higher prevalence of mental disorders, particularly post-traumatic stress disorder (PTSD), but also depression and anxiety disorders in refugees have been reported, compared to the host population [8–10]. As mental disorders are important risk factors for suicide attempt and suicide (suicidal behaviour) [11, 12], the risk can be hypothesised to be elevated in refugees. However, there is limited knowledge regarding this association. Migrant groups are often included in immigrant studies without subclassifying refugee status [13]. Studies that have examined suicidal behaviour specifically in refugees have reported a lower rate of suicide for male refugees, compared to native-born in Denmark [14], and a lower risk for suicide attempt and suicide for refugees, compared to the Swedish-born population [15, 16]. Another Swedish study showed a lower risk for suicide attempt and suicide among refugees with mental disorders, compared to Swedish-born with mental disorders [5]. Refugee minors might be at a higher risk for suicidal behaviour as they are facing the traumatic experiences of a flight in their formative years. Particularly unaccompanied refugee minors lack the social support of a caregiver to help cope with stressful experiences and a difficult acculturation period [6]. However, no previous studies have investigated the risk of suicidal behaviour specifically in young adult refugees, resettling as refugee minors either accompanied or unaccompanied. Neither has the role of mental disorders in the pathway to suicidal behaviour in young refugees been investigated to date.

Besides psychiatric morbidity, socioeconomic status can affect the risk of suicidal behaviour [17, 18]. Young adult refugees tend to have worse school outcomes compared to the general population in the host country and are less likely to attend higher education [19]. It might, therefore, be anticipated that a low educational level has a stronger effect on the risk of suicidal behaviour in refugees than in their majority peers. To date, it is, however, unclear how educational level affects the association between young adult refugees and risk of suicidal behaviour. Due to the documented association between several socio-demographic factors, including labour market marginalisation (LMM), and both refugee status and suicidal behaviour, it is crucial to consider these determinants in analyses of the risk of suicidal behaviour in young refugees [20].

## Aims

We aimed to determine the relationship between young refugees, those who came to Sweden as accompanied or unaccompanied refugee minors, and subsequent risk for suicidal behaviour, compared to the Swedish-born population. Furthermore, a secondary aim was to explore the moderating role of educational level and history of mental disorders in these associations.

## Materials and methods

### Study population

The study base comprised individuals who were 19–30 years old, residing in Sweden on December 31st, 2009 (*n* = 1,425,496). Individuals were excluded if they had incomplete information on reason for settlement in Sweden (*n* = 52,127). Moreover, non-refugee immigrants (*n* = 152,641) and those who had missing information on the year of immigration to Sweden (*n* = 388) were excluded. Additionally, those who came to Sweden when aged 18–30 years (*n* = 19,387) were not included, as the study focused on refugees who came to Sweden as minors. The final study population included 1,200,953 individuals, of whom 51,098 (4.25%) came to Sweden as refugee minors, i.e. below the age of 18. A flow chart illustrates the selection steps to reach the final study population (Supplementary Fig. 1).

### Data sources

The data sources used in this study were Swedish population registers that were linked by an anonymised personal identification number. From Statistics Sweden, the Longitudinal Integration Database for Health Insurance and Labour Market Studies (LISA) provided information on socio-demographics such as sex, age, type of residential area, family situation and educational level. LISA also provided information on LMM characteristics, including number of days with unemployment, number of net days with sickness absence and disability pension. STATIV (The Longitudinal Database for Integration Studies), also from Statistics Sweden, comprised information on reason for residence, i.e. refugee status. From the National Board of Health and Welfare, The National Patient Register (NPR) included information on date and diagnosis regarding in- and specialised outpatient healthcare, coded according to the International Classification of Diseases (ICD-10) [21]. The Causes of Death Register, also from the National Board of Health and Welfare, provided information on date and cause of death [22].

### Exposure measure

The Swedish Migration Agency follows European Union regulations, Swedish legislation and the United Nations Convention relating to the status of refugees regarding granting residence permit in Sweden [23]. Those individuals who were grouped as “refugees” had either of the following indicated for their reason for residing in Sweden: “refugee”, “in need of protection” or on “humanitarian grounds” [24].

Young refugees who arrived as minors (< 18 years) in Sweden and who had at least one accompanying parent at the time of arrival were considered as accompanied refugee minors [25]. To identify refugees who were accompanied by at least one parent at the time of their arrival in Sweden, we used register data on their parents’ residency status and year of immigration. The young refugees were classified as ‘accompanied refugee minors’ if at least one of their parents’ has migrated to Sweden in the same year or any year before the young refugees arrived in Sweden. Those who did not meet these criteria were classified as ‘unaccompanied refugee minors’. Among the 51,098 young refugees in this study, 46,505 (91%) and 4593 (9%) were accompanied and unaccompanied refugee minors, respectively. All individuals born in Sweden were identified as “Swedish-born”.

### Outcome

The main outcome was hospitalisation due to suicide attempt. The secondary outcome was suicide. Suicide attempts were defined as having a diagnosis from hospital admissions during follow-up, coded with ICD-10 codes X60–X84 (self-harm) or Y10–Y34 (events of undetermined intent), retrieved from the NPR. The cases belonging to “undetermined intent” were included to reduce underreporting and to guarantee coherence regarding case ascertainment [26]. Suicide was identified through the ICD-10 codes X60–X84 or Y10–Y34 in the Cause of Death Register.

### Covariates

Socio-demographic covariates included age, sex, educational level, type of residential area, and family situation. They were measured at baseline, January 31st, 2009. LMM factors included unemployment, sickness absence and disability pension, which were all measured during the baseline year (2009). Health-related factors were dichotomised and included mental disorders, with a history of any main or secondary diagnosis with any ICD-10 code ‘F’ in inpatient or specialised outpatient healthcare. Health-related factors also included history of (at least one) hospitalisation due to suicide attempt, and lastly, history of inpatient or specialised outpatient healthcare due to somatic illness from a main or secondary somatic diagnosis with any ICD-10 code (except ‘F’, ‘O’, ‘P’ and ‘Q’ codes). All health-related factors were measured during 2005–2009 and this time period was chosen to use the available data in the best possible way. All covariates were categorised, as seen in Table 1, with missing values shown as separate categories for covariates with missing information.

**Table 1:** Socio-demographic, labour market marginalisation and health-related characteristics of the study cohort of all individuals aged 19–30 years and residing in Sweden on 31 Dec 2009 with a Swedish-born or refugee background (*n* = 1,200,953)

	Swedish-born *n* (column %)	Refugees *n* (column %)	
		Unaccompanied	Accompanied
All (row %)	1,149,855 (95.7)	4593 (0.4)	46,505 (3.9)
Socio-demographic variables (2009)			
Sex			
Women	558,302 (48.5)	1727 (37.6)	22,033 (47.4)
Men	591,553 (51.4)	2866 (62.4)	24,472 (52.6)
Age (years)			
19–24	621,559 (54.1)	2688 (58.5)	26,369 (56.7)
25–30	528,296 (45.9)	1905 (41.5)	20,136 (43.3)
Educational level (years)			
Compulsory school (0–9)	130,019 (11.3)	1580 (34.4)	9024 (19.4)
High school (10–12)	648,476 (56.4)	1678 (36.5)	23,425 (50.4)
College or university (> 12)	358,504 (31.2)	667 (14.5)	12,853 (27.6)
Missing information	12,856 (1.1)	668 (14.5)	1203 (2.6)
Family situation			
Married/cohabiting without children living at home	25,292 (2.2)	274 (6.0)	2741 (5.9)
Married/cohabiting with children living at home	145,156 (12.6)	735 (16.0)	7174 (15.4)
Single without children living at home	956,759 (83.2)	3330 (72.5)	35,390 (76.1)
Single with children living at home	22,648 (2.0)	254 (5.5)	1200 (2.6)
Type of residential area^a^			
Big cities	433,758 (37.7)	2421 (52.7)	21,065 (45.3)
Medium-sized cities	430,174 (37.4)	1541 (33.5)	18,809 (40.4)
Small cities/villages	285,923 (24.9)	631 (13.7)	6631 (14.3)
Labour market marginalisation factors (2009)			
Unemployment between 1 and 180 days^b^	202,288 (17.6)	1153 (25.1)	10,592 (22.8)
Unemployment > 180 days^b^	26,962 (2.3)	315 (6.9)	2330 (5.0)
Sickness absence between 1 and 90 net days^c^	47,858 (4.2)	166 (3.6)	1823 (3.9)
Sickness absence > 90 net days^c^	11,464 (1.0)	44 (0.96)	468 (1.0)
Disability pension in 2009^d^	30,525 (2.6)	117 (2.5)	1120 (2.4)
Health-related factors (2005–2009)			
Inpatient or specialised outpatient healthcare for mental disorders			
Any mental disorder^e.f^	108,284 (9.4)	537 (11.7)	3937 (8.5)
Post-traumatic stress disorder (PTSD)	1930 (0.2)	96 (2.09)	271 (0.6)
Depressive disorders	36, 515 (3.2)	183 (4.0)	1124 (2.4)
Bipolar disorders	5140 (0.4)	18 (0.4)	103 (0.2)
Stress-related disorders	15,926 (1.4)	148 (3.2)	821 (1.8)
Other mental disorders	64,658 (5.6)	261 (5.68)	2045 (4.40)
Inpatient or specialised outpatient somatic healthcare^g.h^	732,721 (63.7)	3061 (66.6)	30,987 (66.6)
Inpatient healthcare due to suicide attempt	10,565 (0.9)	70 (1.5)	383 (0.8)
Outcome (2010–2016) (rate per 100,000 person-years)			
Inpatient healthcare due to suicide attempt	10,078 (127.8)	47 (152.7)	314 (98.8)
Death by suicide	1564 (19.7)	< 10^i^	33 (10.3)

^a^Type of residential area: big cities—Stockholm, Gothenburg and Malmö; medium sized cities—cities with more than 90,000 inhabitants within 30 km distance from the city; small cities/villages

^b^No unemployment group is not presented

^c^No sickness absence group is not presented

^d^No disability pension group is not presented

^e^Mental disorders; any main or secondary diagnosis with the International Classification of Diseases (ICD-10) ‘F’ code was considered ‘yes’ and ‘no’ otherwise

^f^No mental disorder is not presented

^g^Somatic healthcare; any main or secondary diagnosis with neoplasms, diabetes mellitus, diseases of the nervous system, diseases of the circulatory system, disease of the respiratory system, disease of the digestive disorders, measured with International Classification of Diseases (ICD-10) codes

^h^No somatic healthcare is not presented

^i^Number of suicides is fewer than ten and is not reported to minimise the risk of backward identification

Labour market factors were also considered as possible confounders, with adjustments made for unemployment, sickness absence and disability pension. Refugees are more likely to be unemployed compared to the general population [19, 27]. Health-related factors were treated as possible confounders, with adjustments made for previous history of somatic disorders, previous history of mental disorders and previous history of hospitalisation for suicide attempt. History of mental disorders is more common among refugees compared to the general population [10], and rates for suicide attempt were reported to be lower among refugees compared to Swedish-born [28].

### Statistical analysis

Descriptive statistics with frequencies and percentages were calculated for accompanied refugee minors, unaccompanied refugee minors and the Swedish-born population (Table 1). Cox proportional hazard regression models were used to estimate multivariate-adjusted hazard ratios (HR) with 95% confidence intervals (CI) for suicide attempt and suicide in accompanied and unaccompanied refugee minors compared to Swedish-born. Individuals were followed up from baseline, January 1st, 2010, until whichever of the following came first; death, outcome event, emigration or end of follow-up (December 31st, 2016). A Kaplan–Meier survival estimator was used to test the proportional hazard assumption, which was found to be met. Four regression models were applied to adjust for the covariates (Model 1 adjusting for age and sex; Model 2 additionally adjusting for other socio-demographic covariates; Model 3 adjusting for the covariates in model 2 and the LMM factors; Model 4 adjusting for the covariates in model 3 and the health-related factors.

An interaction test, i.e. partial likelihood ratio test was conducted to test the interaction between educational level and refugee status regarding risk of subsequent suicide attempt where educational level was entered as a categorised variable in the model (four categories: compulsory school (0–9 years), high school (10–12 years), college or university (> 12 years) and one category with missing information). Following the same method, interaction between history of mental disorder (Yes, No) and refugee status regarding risk of subsequent suicide attempt was also tested. All mentioned analyses were also performed for suicide as outcome measure. For all analyses a *p* value < 0.05 was considered statistically significant. All statistical analyses were performed using SAS 9.4 for Windows, besides the partial likelihood ratio test which was conducted using SPSS version 25.

### Sensitivity analyses

A sensitivity analysis was performed where those with “in need of protection” or on “humanitarian grounds” were excluded from the refugee category. Another sensitivity analysis was conducted where cases of ‘undetermined intent’ were not included for comparison to the main analysis. Finally, we conducted a sensitivity analysis to investigate the influence of migration-related factors (country of birth and age at arrival) on the risk of suicidal behaviour for unaccompanied refugees compared with accompanied refugees (reference category). As these variables are not applicable for the Swedish-born, they were not included in this analysis as the reference group.

### Ethics

Ethical approval was obtained from the Regional Ethical Review Board, Karolinska Institutet, Stockholm, Sweden (Dnr: 2007/1762-31).

## Results

### Baseline characteristics

The lowest proportion of women (37.6%) was found in the unaccompanied group. In other groups, the proportion of men and women was quite similar. A similar age distribution was seen in all groups. Unaccompanied minors were most likely to have a low educational level (34.4%) and the Swedish-born (31.2%) were most likely to have a high educational level. There were more Swedish-born individuals who were single with or without children than each of the refugee groups (Table 1). Nearly half of the accompanied refugee group (53%) was living in big cities, compared to 38% among the Swedish-born.

Regarding the LMM factors, both refugee groups had higher proportions of unemployed compared to the Swedish-born (Table 1). For the health-related factors, a larger proportion of the unaccompanied group had a history of inpatient or specialised outpatient healthcare for PTSD compared to the Swedish-born (2.1% vs. 0.2%, respectively). Unaccompanied minors had also a higher proportion of stress-related diagnoses compared to the other groups and they had higher incidence rates of subsequent hospitalisation due to suicide attempt (152.7 per 100,000 person-year), compared to Swedish-born and accompanied minors (127.8 and 98.8 per 100,000 person-year, respectively). Swedish-born had higher rates of suicide during the follow-up compared to the accompanied refugee group (19.7 vs. 10.3 per 100,000 person-year).

The distribution of country of birth and age at arrival (years) varied considerably between unaccompanied and accompanied refugee minors. Most unaccompanied refugees came from Iraq and Somalia (28.2% and 17.5%, respectively). On the other hand, almost half of the accompanied refugees came from former Yugoslavia (Supplementary Table S1). Among unaccompanied refugees, 45% arrived in Sweden at 17+ years of age and the corresponding proportion for accompanied refugees was 5%.

### Risk of suicide attempt and suicide

Table 2 presents the risk of suicide attempt and suicide during follow-up in refugee minors compared to Swedish-born. Refugee minors had an around 20% lower risk of suicide attempt in the fully adjusted analysis compared to Swedish-born. The accompanied refugee group had a statistically significant lower risk for suicide attempt compared to the Swedish-born (Model 4 HR 0.77, 95% CI 0.69–0.87). In the fully adjusted models, the risk for suicide attempt for unaccompanied refugee minors showed non-significant results (HR 0.83, 95% CI 0.62–1.10). Refugee minors had a significantly lower risk of suicide (Table 2). For the unaccompanied group, there were less than ten cases and the results were not statistically significant. For the accompanied group, the risk of suicide was statistically significant, and 46% lower compared to Swedish-born in the fully adjusted model.

**Table 2:** Risk of suicide attempt and suicide during 2010–2016 in young adult refugees who resettled as unaccompanied or accompanied refugee minors in comparison with the Swedish-born population, multivariate hazard ratios (HR) with 95% confidence intervals (CI)

	*n* (rate per 100,000 person-years)	Model 1^a^ HR (95% CI)	Model 2^b^ HR (95% CI)	Model 3^c^ HR (95% CI)	Model 4^d^ HR (95% CI)
Suicide attempt					
Swedish-born	10,078 (127.8)	1 (REF)	1 (REF)	1 (REF)	1 (REF)
Refugees	361 (103.5)	**0.81 (0.72–0.90)**	**0.65 (0.59–0.72)**	**0.68 (0.61–0.75)**	**0.78 (0.70–0.87)**
Unaccompanied	47 (152.7)	1.20 (0.90–1.60)	**0.70 (0.52–0.93)**	0.79 (0.60–1.06)	0.83 (0.62–1.10)
Accompanied	314 (98.8)	**0.77 (0.69–0.86)**	**0.65 (0.58–0.72)**	**0.66 (0.59–0.74)**	**0.77 (0.69–0.87)**
Suicide					
Swedish-born	1564 (19.7)	1 (REF)	1 (REF)	1 (REF)	1 (REF)
Refugees	40 (11.4)	**0.57 (0.41–0.78)**	**0.50 (0.36–0.68)**	**0.51 (0.37–0.70)**	**0.57 (0.41–0.78)**
Unaccompanied	< 10^e^ (22.6)	1.04 (0.50–2.19)	0.75 (0.35–1.57)	0.80 (0.38–1.68)	0.83 (0.39–1.75)
Accompanied	33 (10.3)	**0.52 (0.37–0.73)**	**0.47 (0.33–0.66)**	**0.47 (0.33–0.67)**	**0.54 (0.38–0.76)**

HRs with 95% CIs in bold indicate statistically significant association (*p* value < 0.05)

^a^Model 1: adjusted for age and sex

^b^Model 2: adjusted for model 1 covariates and other socio-demographic factors (education, family situation and type of residential area)

^c^Model 3: adjusted for model 2 covariates and labour market marginalisation factors (unemployment in 2009 (0, 1–180 days, > 180 days), sickness absence in 2009 (0, 1–90 days, > 90 net days) and disability pension in 2009 (yes, no)

^d^Model 4: adjusted for model 3 covariates and health-related factors (history of inpatient or specialised outpatient healthcare for mental disorders in 2005–2009, history of inpatient or specialised outpatient somatic healthcare in 2005–2009 and history of hospitalisation due to suicide attempt 2005–2009)

^e^Number of suicides is fewer than ten and is not reported to minimise the risk of backward identification

### Risk of suicidal behaviour by educational level

Compared to Swedish-born with high educational level, Swedish-born with low educational level had a significantly higher risk for suicide attempts (HR 11.71, 95% CI 10.91–12.55). After adjusting for the LMM factors, the risk estimate decreased mostly because of disability pension (HR 8.3, 95% CI 7.7–8.94), and with further adjustments, the HR decreased primarily because of previous history of mental disorder (HR 4.35, 95% CI 4.03–4.69). Refugees with high educational level had a lower risk of suicide attempts compared to the Swedish-born with high educational level (HR 0.63, 95% CI 0.42–0.96). For refugees with low educational level there was a sixfold higher risk (HR 6.51, 95% CI 5.56–7.62) that decreased significantly with the biggest effect from previous history of mental disorder on the risk estimate in the fully adjusted model (Table 3). Results regarding the risk of suicide for refugees showed comparable patterns as those for subsequent suicide attempt (Table 3). The results of the partial likelihood ratio test revealed a non-significant interaction between educational level and refugee status regarding subsequent suicide attempt in the crude model (*p* value 0.177) and multivariate model (*p* value 0.621) (data not shown). This interaction was also non-significant in relation to subsequent suicide in the crude model (*p* value 0.938) and in the multivariate model (*p* value 0.997) (data not shown).

**Table 3:** Risk of suicide attempt and suicide during 2010–2016 in Swedish-born individuals and young adult refugees by educational level, multivariate hazard ratios (HR) with 95% confidence intervals (CI)

	*n* (rate per 100,000 person-years)	Model 1^a^ HR (95% CI)	Model 2^b^ HR (95% CI)	Model 3^c^ HR (95% CI)	Model 4^d^ HR (95% CI)
Suicide attempt					
Educational level of Swedish-born					
High	972 (39.5)	1 (REF)	1 (REF)	1 (REF)	1 (REF)
Medium	4795 (107.6)	**2.81 (2.62–3.01)**	**2.79 (2.60–2.99)**	**2.34 (2.18–2.51)**	**2.05 (1.91–2.20)**
Low	4073 (462.9)	**12.24 (11.40–13.15)**	**12.18 (11.33–13.09)**	**8.30 (7.70–8.94)**	**4.35 (4.03–4.69)**
Missing	238 (276.0)	**7.25 (6.29–8.36)**	**6.89 (5.98–7.95)**	**2.41 (2.07–2.81)**	**2.19 (1.88–2.55)**
Educational level of refugees					
High	23 (24.9)	**0.63 (0.41–0.95)**	**0.64 (0.43–0.97)**	**0.62 (0.41–0.93)**	**0.63 (0.42–0.96)**
Medium	133 (77.4)	**2.03 (1.69–2.43)**	**2.12 (1.77–2.55)**	**1.70 (1.42–2.04)**	**1.56 (1.30–1.88)**
Low	185 (257.5)	**6.89 (5.88–8.06)**	**7.22 (6.17–8.47)**	**5.23 (4.46–6.13)**	**3.50 (2.98–4.11)**
Missing	20 (158.4)	**4.14 (2.66–6.44)**	**4.16 (2.67–6.48)**	**2.58 (1.66–4.03)**	**2.03 (1.30–3.16)**
Suicide					
Educational level of Swedish-born					
High	203 (8.2)	1 (REF)	1 (REF)	1 (REF)	1 (REF)
Medium	795 (17.8)	**2.07 (1.77–2.42)**	**2.09 (1.79–2.46)**	**1.80 (1.53–2.11)**	**1.60 (1.36–1.87)**
Low	549 (61.3)	**6.90 (5.85–8.13)**	**7.02 (5.94–8.29)**	**4.99 (4.19–5.93)**	**2.73 (2.29–3.26)**
Missing	17(19.5)	**2.23 (1.36–3.67)**	**2.10 (1.28–3.45)**	0.79 (0.47–1.33)	0.72 (0.43–1.21)
Educational level of refugees					
High	< 10^e^ (4.3)	0.54 (0.20–1.45)	0.56 (0.21–1.50)	0.54 (0.20–1.45)	0.55 (0.21–1.49)
Medium	16 (9.3)	1.06 (0.64–1.77)	1.16 (0.70–1.93)	0.95 (0.57–1.58)	0.88 (0.53–1.47)
Low	19 (26.2)	**2.88 (1.80–4.62)**	**3.18 (1.98–5.10)**	**2.38 (1.48–3.82)**	1.60 (1.00–2.58)
Missing	< 10^e^ (7.9)	0.94 (0.13–6.72)	0.97 (0.14–6.89)	0.62 (0.09–4.42)	0.47 (0.07–3.37)

HRs with 95% CIs in bold indicate statistically significant association (*p* value < 0.05)

^a^Model 1: adjusted for age and sex

^b^Model 2: adjusted for model 1 covariates and other socio-demographic factors except education (family situation and type of residential area)

^c^Model 3: adjusted for model 1 covariates and labour market marginalisation factors (unemployment in 2009 (0, 1–180 days, > 180 days), sickness absence in 2009 (0, 1–90 days, > 90 net days) and disability pension in 2009 (yes, no)

^d^Model 4: adjusted for model 2 covariates and health-related factors (history of inpatient or specialised outpatient healthcare for mental disorders in 2005–2009, history of inpatient or specialised outpatient somatic healthcare in 2005–2009 and history of hospitalisation due to suicide attempt 2005–2009)

^e^Number of suicides is fewer than ten and is not reported to minimise the risk of backward identification

### Previous history of mental disorder and risk of suicidal behaviour

Table 4 presents the results for the analysis of the risk of suicide attempt and suicide by previous history of mental disorder. Swedish-born with a history of mental disorders had a 13-fold higher risk of suicide attempts (Model 1—Table 4) compared to Swedish-born without a history of mental disorders. Adjusting for socio-demographic covariates, particularly for educational level, had the strongest effect on the risk estimate which decreased from 13.00 in Model 1 to 9.22 in Model 2. The risk was statistically significant and sixfold higher in the fully adjusted model and decreased mostly due to previous history of hospitalisation for suicide attempt (Model 3—Table 4).

**Table 4:** Risk of suicide attempt and suicide in Swedish-born individuals and refugees by previous history of mental disorder^a^, multivariate hazard ratios (HR) with 95% confidence intervals (CI)

	*n* (rate per 100,000 person-years)	Model 1^b^ HR (95% CI)	Model 2^c^ HR (95% CI)	Model 3^d^ HR (95% CI)	Model 4^e^ HR (95% CI)
Suicide attempt					
History of mental disorder in Swedish-born					
No	4337 (60.5)	1 (REF)	1 (REF)	1 (REF)	1 (REF)
Yes	5741 (797.0)	**13.00 (12.49–13.52)**	**9.22 (8.84–9.62)**	**7.99 (7.64–8.35)**	**5.94 (5.67–6.23)**
History of mental disorder in refugees					
No	194 (60.9)	1.00 (0.86–1.52)	0.89 (0.77–1.03)	0.87 (0.75–1.00)	**0.86 (0.74–0.99)**
Yes	167 (557.3)	**9.13 (7.83–10.66)**	**6.24 (5.34–7.29)**	**5.47 (4.68–6.40)**	**4.20 (3.59–4.91)**
Suicide					
History of mental disorder in Swedish-born					
No	762 (10.6)	1 (REF)	1 (REF)	1 (REF)	1 (REF)
Yes	802 (107.8)	**11.22 (10.16–12.40)**	**8.75 (7.87–9.73)**	**7.77 (6.94–8.69)**	**6.34 (5.62–7.14)**
History of mental disorder in refugees					
No	23 (7.2)	0.67 (0.44–1.01)	**0.65 (0.43–0.98)**	**0.63 (0.42–0.96)**	**0.63 (0.42–0.96)**
Yes	17 (55.5)	5.32 (3.29–860)	**4.21 (2.59–6.82)**	**3.75 (2.31–6.09)**	**3.19 (1.97–5.19)**

HRs with 95% CIs in bold indicate statistically significant association (*p* value < 0.05)

^a^Mental disorder; any main or secondary diagnosis with the International Classification of Diseases (ICD-10) ‘F’ code (during 2005–2009) was considered ‘yes’ and ‘no’ otherwise

^b^Model 1: adjusted for age and sex

^c^Model 2: adjusted for Model 1 covariates and other socio-demographic factors (education, family situation and type of residential area)

^d^Model 3: adjusted for model 2 covariates and labour market marginalisation (LMM) factors (unemployment in 2009 (0, 1–180 days, > 180 days), sickness absence in 2009 (0, 1–90 days, > 90 net days) and disability pension in 2009 (yes, no)

^e^Model 4: adjusted for model 3 covariates and health-related factors (history of inpatient or specialised outpatient somatic healthcare in 2005–2009 and history of hospitalisation due to suicide attempt 2005–2009)

Refugees without a history of mental disorder had a significantly lower HR (0.86) regarding subsequent suicide attempt in the fully adjusted model (Table 4). The analysis for refugees with a history of mental disorders showed a statistically significant ninefold higher risk of suicide attempt compared to Swedish-born without mental disorder. The risk was fourfold in the fully adjusted model with educational level, unemployment and previous history of hospitalisation for suicide attempt as the covariates with the strongest effect (Table 4). Patterns regarding the association of refugee status and subsequent suicide stratified for previous mental disorder resembled those for suicide attempt (Table 4).

The results of the partial likelihood ratio test showed a significant interaction of previous history of mental disorders with refugee status regarding subsequent suicide attempt in the crude model (*p* value 0.001) (data not shown), however, the interaction was non-significant in the multivariate model (*p* value 0.069). This interaction was not significant concerning subsequent suicide in the crude model (*p* value 0.391) or the multivariate model (*p* value 0.482) (data not shown).

### Sensitivity analyses

The results, excluding those with “in need of protection” or on “humanitarian grounds, showed that the risk for suicide attempt among young refugees was lower compared to their Swedish-born peers (multivariate-adjusted HR 0.78, 95% CI 0.70–0.87). The risk for suicide was also lower (HR 0.57, 95% CI 0.42–0.78). These results were similar to the findings from our main analysis on young refugees (Table 2). Results from another sensitivity analysis excluding cases with ‘undetermined intent’ in the definition of suicidal behaviour showed similar risk estimates to the main analysis (data not shown). Finally, the change in point estimates between the models with or without the migration-related covariates (country of birth and age at arrival) showed no major influence of these factors on the risk of suicidal behaviour for unaccompanied refugee minors (Supplementary Table S2).

## Discussion

### Main findings

This population-based cohort study showed that refugees aged 19–30 years who came as accompanied minors had a lower risk for suicide attempt and suicide compared to Swedish-born, while the risk for unaccompanied refugee minors did not show statistically significant differences, compared to the Swedish-born. Low educational level and previous mental disorders increased the risk of suicidal behaviour in both Swedish-born and refugees, but risk estimates in refugees continued to be lower.

### Young adult refugees and suicidal behaviour

As there are no previous studies on young adult refugees resettling in a Westernised host country as minors and their subsequent risk of suicidal behaviour, it is challenging to make a direct comparison. However, recent Swedish studies found a lower risk of suicide attempt and suicide during follow-up for refugees (all ages) compared to the Swedish-born population [16, 28]. Moreover, a longitudinal study in Denmark with 12 years of follow-up showed a lower rate ratio (RR 0.38, 95% CI 0.24–0.61) of suicide for male refugees (all ages) compared with a Danish-born comparison group [14]. In Ontario, Canada, adult refugees had lower crude rates of self-harm (8.1 vs. 9.7/100,000 person-years) and suicide (6.1 vs. 11.8/100,000 person-years) compared with the host population, during an observation period of 15 years [29]. As, both studies included all individual aged 18 years and above, they are not directly comparable with our study cohort. Several studies have reported higher rates of mental disorders among refugees, though [8, 9]. Therefore, the result showing a lower risk of suicidal behaviour for former refugee minors does not align with the existing literature regarding mental disorders being the strongest factor for suicidal behaviour [11]. A few possible explanations might contribute to these results. A phenomenon called “The healthy migrant effect” has been discussed in the literature, which is a theory arguing that some migrants have better health compared to the general population in the host country [14]. The refugees who could obtain refugee status in Sweden can, therefore, be a selected group because some refugees with poorer mental health may not have overcome the complicated process of migrating to another country. A cultural aspect could also be protective, as many refugees come from Islamic countries where suicide is culturally not acceptable and thus, according to a cultural perspective of suicidal behaviour, this could affect how individuals from Islamic countries react to stressful events and to suicidal ideation [30]. Moreover, differences in adverse health behaviour might matter. Increased alcohol intake, mostly through acute alcohol use, is known to be associated with suicidal behaviour [31, 32]. It has been reported that Muslims drink less alcohol compared to the general population [33], which could partly explain the association found in this study. Our sensitivity analysis showed that country of birth had no major influence on the risk of suicidal behaviour for unaccompanied refugees, compared with accompanied refugees. This may suggest that apart from the cultural aspects, other factors such as resiliency of young refugees may explain their lower risk of suicidal behaviour.

The lower risk of suicidal behaviour found in young adult refugees who resettled in Sweden as accompanied minors, could, however, not be found for those entering Sweden unaccompanied by a caregiver as the result was not statistically significant, in part due to lack of statistical power. It has been shown that the support of one’s family, and other important social connections, strengthen the resilience and coping strategies of vulnerable children [34]. Due to this, the post-migration period can be particularly stressful for unaccompanied refugee minors, as they lack parental guidance that can give protection for new challenges and old traumatic experiences [35]. Previous reports suggest also higher rates of PTSD in young refugees previously resettling in Sweden as unaccompanied minors and unaccompanied minors seeking asylum in Sweden [36, 37]. Future studies with adequate statistical power to perform analyses on suicide in unaccompanied minors are needed to draw firm conclusions regarding their risk of suicidal behaviour.

Socioeconomic status measured as educational level had a strong modifying role in the association with subsequent suicidal behaviour for both refugees and Swedish-born. A low educational level has previously been shown to be an important factor associated with suicidal behaviour [11]. Socioeconomic status is also an important parameter regarding health measures of young immigrants and immigrant children, and advances in education is considered being a factor that can lead directly to wellbeing and better health [38]. Furthermore, a Swedish study has shown that refugees with higher education seek more psychiatric care than those with low education [19]. Still, our findings suggest that young adult refugees have a suicide attempt risk which is closer to that of the Swedish-born if both have a low socioeconomic status, than the relative comparison between refugees and Swedish-born with high education. These findings need further evaluation, particularly regarding the pathways to suicidal behaviour in young refugees with low academic achievements.

Similarly, to educational level, mental disorders also had a strong association with subsequent suicidal behaviour among both young refugees and Swedish-born, but the risk for suicidal behaviour was relatively lower in refugees with mental disorders. Comparable findings for young refugees are not available, but similar results were found in a recently published study on refugees of all ages [28]. It showed that refugees have a higher prevalence of mental disorders compared to Swedish-born, and when comparing refugees and Swedish-born with a history of mental disorders, refugees had lower rates and risk of suicide attempt [28]. Our findings are in line with this study. Still, the relative excess risk difference between refugees and Swedish-born with mental disorders seems to be lower in our study focusing on young adults compared to the mentioned Swedish study on all age groups. While these findings need replication, possible reasons for age differences in the importance of mental disorders regarding subsequent suicidal behaviour in refugees and Swedish-born might be due to age-dependent peculiarities of suicidal behaviour and help-seeking due to mental disorders [39–41]. Although, all individuals with refugee status have the same legal access to healthcare and welfare services as the Swedish-born majority, there are multiple barriers that may hinder the access to these services for refugees such as language difficulties, limited knowledge of how the healthcare and welfare system function in Sweden etc. [42] Moreover, culture-bound views on mental ill-health and suicidal behaviour may vary considerably between young refugees and their Swedish-born peers, which influences the differences in help-seeking due to any mental disorder between these groups [43].

### Methodological considerations

The strengths of this study include the population-based design with minimal loss to follow-up, data being gathered from nation-wide registers with high completeness [22, 44, 45], and the possibility of adjusting for a range of confounders. The available registers provided here an exceptional possibility to investigate a rare outcome measure (such as suicidal behaviour) in relatively small populations (i.e. young refugees) with a long follow-up period. Here, this study is unique as only a few countries offer the possibility for using nation-wide high-quality registers for research and Sweden has the largest population size of refugees of these countries.

There are also some limitations to be mentioned. First, we lacked information on whether any young refugees came to Sweden accompanied by some other guardian than their parents. There is also a risk of underreporting of suicide attempts as it is possible that only the most serious cases are captured through hospitalisation [46]. It is likely that the less serious cases of suicide attempt among the refugee groups would be underreported due to culture-related stigmatisation. This could lead to a differential misclassification bias that could possibly decrease the estimates. Including cases of undetermined intent might, however, have reduced the possibility of bias in our study. Information on the history of mental disorders was obtained from the NPR with data on inpatient and specialised outpatient healthcare, which might also have led to considering mainly severe cases of mental disorders with the possibility of refugees being underrepresented [45]. A further limitation comprises the phenomenon of reverse causality in the analyses of educational level and subsequent suicidal behaviour, i.e. mental health problems in childhood or in early adolescence could affect ones’ education negatively, e.g. school attendance and educational motivation [47]. To minimise such an effect, adjustments were made for history of mental disorders during the five-year period before the start of the follow-up. Finally, the results of this study could only be generalisable to other high-income countries with comparable health and welfare systems as well as migration regulations as Sweden.

## Conclusion

This study indicates that young adult refugees, who have migrated to Sweden as minors, had a lower risk of suicide attempt and suicide compared to Swedish-born. This was true for those entering Sweden being accompanied by a parent, but no significant differences were observed for those young refugees who entered Sweden without a caregiver. Low educational level and previous mental disorders seem to be important factors regarding risk for suicide attempt, both for Swedish-born and refugees. Young refugees who were not accompanied by a parent when entering Sweden need tailor-made and culturally specific intervention strategies. More scientific knowledge on the pathways to suicidal behaviour in young adult refugees with low education and mental disorders is warranted.

## Supplementary Information

Below is the link to the electronic supplementary material.

Supplementary file 1 (DOCX 40 KB)
