# Supplementary material for: Risk of suicide attempt and suicide in young adult refugees compared to their Swedish-born peers: a register-based cohort study
Source: Soc Psychiatry Psychiatr Epidemiol. 2021 Apr 29;56(12):2163–73. doi: 10.1007/s00127-021-02099-5 (PMC8558272; doi:10.1007/s00127-021-02099-5)
Supplement: Supplementary file 1 — (DOCX 40 KB) [file 127_2021_2099_MOESM1_ESM.docx]

**Supplementary Table S1.** Migration-related characteristics of young adult refugees aged 19-30 years and residing in Sweden on 31 Dec 2009 who resettled as unaccompanied or accompanied refugee minors in Sweden (n=1 200 953).

|  | **Unaccompanied refugee minors** | **Accompanied refugee minors** |
| --- | --- | --- |
| All (row %) | 4 593 (9.0%) | 46 505 (91%) |
| **Migration-related factors (2009)** |  |  |
| Country of birth |  |  |
| Eritrea | 73 (1.6) | 341 (0.7) |
| Ethiopia | 82 (1.8) | 407 (0.9) |
| Somalia | 802 (17.5) | 794 (1.7) |
| Other countries in Africa | 338 (7.4) | 710 (1.5) |
| Afghanistan | 365 (7.9) | 608 (1.3) |
| Iran | 348 (7.6) | 4728 (10.2) |
| Iraq | 1294 (28.2) | 5142 (11.1) |
| Syria | 136 (3.0) | 1445 (3.1) |
| Other countries in Asia | 357 (7.8) | 5430 (11.7) |
| Chile | 67 (1.5) | 1428 (3.1) |
| Other countries in South America | 49 (1.1) | 787 (1.7) |
| Former Yugoslavia | 533 (11.6) | 21 936 (47.2) |
| All other countries | 149 (3.2) | 341 (5.9) |
| Age at arrival (years) |  |  |
| 0-6 | 447 (9.7) | 16715 (35.9) |
| 7-13 | 1249 (27.2) | 22364 (48.1) |
| 14-16 | 838 (18.2) | 5148 (11.1) |
| 17+ | 2059 (44.8) | 2278 (4.9) |

**Supplementary Table S2**. Risk of suicide attempt and suicide during 2010-2016 in young adult refugees who resettled as unaccompanied refugee minors in comparison with accompanied refugee minors, crude and multivariate hazard ratios (HR) with 95% confidence intervals (CI).

|  | **n (rate per 100 000 person-years)** | **Crude HR (95% CI)** | **Model 1^a^ HR (95% CI)** | **Model 2^b^ HR (95% CI)** | **Model 3^c^ HR (95% CI)** | **Model 4^d^ HR (95% CI)** |
| --- | --- | --- | --- | --- | --- | --- |
| **Suicide attempt** |  |  |  |  |  |  |
| Accompanied refugees | 314 (98.8) | 1 (REF) | 1 (REF) | 1 (REF) | 1 (REF) | 1 (REF) |
| Unaccompanied refugees | 47 (152.7) | **1.54 (1.13-2.09)** | 1.12 (0.82-1.54) | 1.19 (0.87-1.62) | 1.09 (0.80-1.50) | 1.07 (0.75-1.53) |
| **Suicide** |  |  |  |  |  |  |
| Accompanied refugees | 33 (10.3) | 1 (REF) | 1 (REF) | 1 (REF) | 1 (REF) | 1 (REF) |
| Unaccompanied efugees | <10^e^ (22.6) | 2.19 (0.97-4.95) | 1.70 (0.74-3.94) | 1.80 (0.78-4.14) | 1.59 (0.68-3.68) | 1.58 (0.60-4.15) |

HRs with 95% CIs in bold indicate statistically significant association (p-value <0.05)

^a^ Adjusted for age and sex, education, family situation and type of residential area

^b^ Adjusted for model 1 covariates and labour market marginalisation factors: unemployment in 2009 (0, 1-180 days, >180 days), sickness absence in 2009 (0, 1-90 days, >90 net days) and disability pension in 2009 (Yes, No)

^c^ Adjusted for model 2 covariates and health-related factors (history of inpatient or specialised outpatient healthcare for mental disorders in 2005-2009, history of inpatient or specialised outpatient somatic healthcare in 2005-2009 and history of hospitalisation due to suicide attempt 2005-2009)

^d^ Adjusted for model 3 covariates and migration-related factors: country of birth and age at arrival (0-6, 7-13, 14-16 and 17+ years)

^e^ Number of suicides is fewer than ten and is not reported to minimise the risk of backward identification

Initial study population (n=1 425 496 individuals):
- All registered residents in Sweden on December 31st, 2009
- 19-30 years of age

n=52 127 individuals were excluded due to incomplete information on reason for settlement in Sweden

n=1 373 369 individuals after initial exclusion who can be categorised into Swedish-born, refugees and non-refugee immigrants based on information on reason for settlement

n=152 641 non-refugee immigrants were excluded

n=1 220 728 individuals after further exclusion of which 1 149 855 Swedish-born and 70 873 refugees

n=388 individuals with missing data on latest year of immigration were excluded

n=1 200 953 individuals after final exclusion of which 1 149 855 Swedish-born, 51 098 refugees (4593 unaccompanied refugee minors and
46 505 accompanied refugee minors)

n=1 220 340 individuals after further exclusion of which 1 149 855 Swedish-born, 70 485 refugees

n=19 387 refugees who came to Sweden as adults (>18 years old) were excluded

Supplementary Fig 1. Flow chart of selection of the study population.
